# Studies on Wear of a Milling Chuck for a Production Line of Specialized Elements Used in Lockstitch Machines

**DOI:** 10.3390/ma15093402

**Published:** 2022-05-09

**Authors:** Marek Wozniak, Tomasz Zadzimski, Adam Rylski, Marcin Makówka, Przemysław Kubiak, Krzysztof Siczek

**Affiliations:** 1 Department of Vehicles and Fundamentals of Machine Design, Lodz University of Technology, 90-537 Lodz, Poland; marek.wozniak.1@p.lodz.pl; 2 Lodz University of Technology, 90-924 Lodz, Poland; 218883@edu.p.lodz.pl (T.Z.); przemyslaw.kubiak@pw.edu.pl (P.K.); 3 Institute of Material Science and Engineering, Lodz University of Technology, 90-537 Lodz, Poland; adam.rylski@p.lodz.pl (A.R.); marcin.makowka@p.lodz.pl (M.M.); 4 Faculty of Automotive and Construction Machinery Engineering, Warsaw University of Technology, 02-524 Lodz, Poland

**Keywords:** wear, lockstitch machine, milling chuck, DLC coating, finite element method

## Abstract

The study aims to determine the wear intensity of selected milling chuck assembly surfaces covered with a protective DLC (Diamond Like Carbon) coating, used on the production line for elements of selected lockstitch machines, and to analyze the stress distributions in the object fixed with such a chuck for the characteristic load systems of this object during its processing. A model of the workpiece was developed using the finite element method. The boundary conditions, including the load and the method of clamping the workpiece, resulted from the parameters of the milling process and the geometric configuration of the milling chuck. Stress distributions in the workpiece for specific milling parameters and for various configurations of the milling chuck holding the workpiece are included in the article. The model experimental studies of wear were conducted in the contact zone between two surfaces covered with DLC: one on the element of the milling chuck pressing the workpiece and the other on the eccentric cams of this holder. The obtained wear values and shapes for the worn surfaces are also shown. The wear intensities for the steel plunger fins modelling swivel arm of the holder were by an order higher than those of corresponding steel shaft shoulders modelling eccentric cam of the holder. The linear wear intensities for these mating components may be expressed in terms of a function of average contact pressure and sliding speed in a corresponding contact zone. The indentation of eccentric cam into mating surface of the swivel arm of the holder increased nonlinearly with the enhancement of number of cycles of the eccentric cam.

## 1. Introduction

The sewing process of various materials in the form of fabrics or knitted fabrics is used in many industries, including fashion (clothes) [1,2,3], medical (protective clothing, bedding) [4,5], tourism and sports (tents and overalls) [6,7,8], military (uniforms) [9], furniture (upholstery) [10], automotive (airbags, seat belts) [11,12], and others. The process is carried out with the use of various sewing machines, including lockstitch machines. The sewing process is accompanied by abrasive wear [13] and more seldom deformations of machine elements, particularly needles [14]. Moreover, it is often necessary to adapt classic machines to the requirements of the specific sewing process. This is done by modifying their components of machines or assembling components [15]. The mentioned modification often requires conducting various cutting operations, e.g., turning, milling, or grinding the surface of the elements.

**Figure 1:**
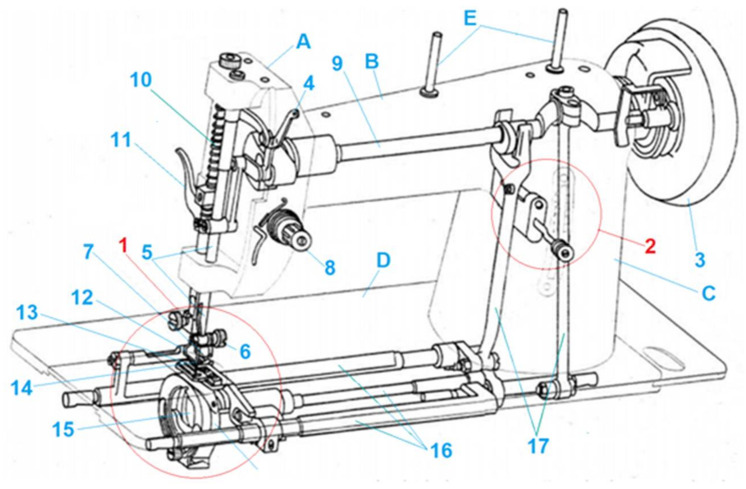
General structure and kinematic system of a lockstitch machine. (A) Sewing Head, (B) Horizontal Arm, (C) Upright Arm, (D) Machine Bed, (E) Spool Pin, (1) Stitch creation mechanism, (2) Stitch adjustment mechanism, (3) Machine pulley, (4) Thread take-up lever, (5) Needle bar, (6) Needle clam screw, (7) Needle holder (8) Needle thread tension assembly, (9) Arm shaft, (10) Pressure bar, (11) Pressure bar lifter, (12) Pressure foot, (13) Needle, (14) Feed dog, (15) Bobbin case, and (16) Loop-taker (Shuttle), (17) Driving Arm.

The aim of the present study is to investigate the intensity of wear of selected milling chuck assembly surfaces covered with a protective diamond-like coating (DLC), used on the production line for elements of selected lockstitch machines, and to analyze the stress distributions in the object fixed with such a chuck for the characteristic load systems of this object during its processing. This workpiece was the bobbin case of the first of the possible types of lockstitches including one needle (straight) and double needle (zigzag) [16]. Figure 1 shows the general structure and kinematic system of a one needle lockstitch machine. Such machines comprise of a head, base, drive, and several various mechanisms and devices facilitating and accelerating sewing work [16]. The main task of a lockstitch machine is to create a stitch. A formation of a latter and the interaction of the components and variables affecting a ‘good seam’ is presented in [17]. The evolution of the lockstitch machine has been discussed in [18]. Various characteristics of lockstitch machine and work aids for best sewing performance are discussed in [19]. The technological development of sewing garments was presented in [20]. Interestingly, Stylios [21] applied a neural network, utilizing sewing machine interactions at various speeds to formulate qualitative rules, and mapping fabric properties to optimum sewing machine settings for intelligent sewing machines.

During operation, the sewing machines can be noisy. Beran et al. [22] experimentally studied the noise generation during the operation of various mechanisms of sewing machines. They found that the maximum value of the level of sound power can reach 83.6 dB.

The structure of the proper stitch obtained by lockstitch was presented in [23].

The manufacturing process of sewing machines with various degrees of shape complication requires the use of various technologies, including casting and machining. The latter is realized with the use of 3 and 5-axis CNC (Computer Numerical Control) milling machines.

Such a design of the machined surfaces has a positive effect on the repeatability of the positions of the actuators attached to them, the bases of sensors of the sewing machine mechanisms, both in relation to the reference databases of the machine tool, and such attached elements in relation to each other. Lockstitch machines with complex structures equipped with electronic devices are used to produce automotive airbags.

During processing, the bobbin case is pressed to the bottom fixing surfaces of the milling chuck by various components of such a chuck. Realization of such components has evolved with the design on the chuck. The latter version utilized the eccentricity, pressing a hinged plate holding the workpiece. The eccentricity and hinged plate were the integral components of the chuck. All surfaces of such components were covered by DLC layers. Multiple pressing and releasing of the eccentric with this plate took place under conditions of changing load and slip values, leading to wear of their mating surfaces. The value of this wear and the shape of the worn zones were determined because of the experimental tests conducted in the model of such contact under load and slip conditions close to reality. The obtained wear results in the model were used to estimate the contact wear of the mating surfaces of the actual milling chuck, covered with a DLC protective layer.

During the machining process of the bobbin case positioned in the milling chuck, the stress distribution occurs, affected by the milling parameters and geometric parameters of the milling chuck and the method of positioning the workpiece in it. Using the finite element method (FEM), a model of the bobbin case was elaborated. The boundary conditions, including the load and the method of clamping the workpiece, resulted from the parameters of the milling process and the geometric configuration of the milling chuck. The analysis of the stress distribution was assumed to be in steady state conditions, under mean values of cutting forces in the contact of the cutter and the machined surface along whole machined surfaces.

## 2. DLC Coatings for Various Components, Particularly Made of Steel

Nowadays, the application of DLC coatings is very wide, particularly for various elements made of steel, from which components of the milling chuck analyzed were made. 

Rajak et al. [24] noticed that DLC coatings are utilized in mechanical, civil, aero space, automobile, biomedical, marine, and several other manufacturing industries. The coating life of each DLC is affected by its constituent elements and manufacturing techniques.

Using ion beam technology, Aisenberg and Chabot [25] obtained layers exhibited diamond-like features for the first time. Due to their high hardness, modulus of elasticity, and resistivity accompanied by the chemical inertness close to the diamond, they started being named the DLC ones.

The DLC layers are widely applied for various surface-protective solutions [26,27,28,29]. 

DLC coatings can be either doped: W-DLC [30,31,32,33], Cr-DLC [34], Si-DLC [35]; or non-doped: a-C:H [34,35,36], ta-C [35]. 

Tuszynski et al. [37] noticed that DLC coatings are most often evaluated for their tribological properties.

The doped-DLC coatings exhibited higher resistance to wear, good adhesion with the substrate, increased electrical conductivity, and weakened compressive internal stresses during deposition [38,39,40,41,42,43].

Interestingly, the DLC layers were found chemically inert [38,44,45]. 

The mechanical and tribological properties of DLC films are highly influenced by the ratio of sp2 to sp3 hybridized carbon bonds and hydrogen content [46,47]. The lower values of this ratio provided the higher mechanical properties of the film, whereas the higher values of it allows low coefficient of friction and lower mechanical properties of the film. 

According to [48], the DLC coatings exhibit low coefficients of friction.

According to [38], depending on H content and sp3 bonds, DLC coatings can be classified as “soft” or “hard” films. 

The resistance to wear of the DLC layer under dry friction conditions can be less than 10^−8^ mm^3^N^−1^m^−1^, while keeping low friction coefficient (0.005–0.5) [49]. Both emerged from the graphitization process which occurred in the contact area between mating elements. The graphitization process was more intense for higher loads and sliding speeds [49]. The mechanical and tribological properties of carbon coatings were also affected by the hydrogen content. Stabilizing the σsp^3^ hybridized carbon structures, hydrogen prevented their unwanted transformation into the graphitic phase. The strong chemical affinity of carbon for hydrogen blocked the adhesive interactions of free σ bonds with the substrate material or particles of oxygen, water vapor, or working gases in the atmosphere. It has usually enhanced the friction coefficient. However, very weak values of Van der Waals forces between the surface layers of hydrogen allowed lower values of friction coefficient to be reached than those observed in the case of amorphous non-hydrogenated carbon layers [50].

However, DLC coatings exhibited an important level of residual stress which enhanced with hardness and overall thickness of the film. Such a residual stress may induce and cause cracking of the coating. The DLC self-delamination may occur only because of generated stress, with values reaching up to 10 GPa [49,51].

The applications of various DLC coatings were discussed by Moriguchi et al. in their review [52]. Particularly, the DLC coatings are beneficial for various combustion engines components [46,53,54,55,56,57,58,59,60,61,62,63,64,65,66,67].

Using various tribotesters, the tribological properties of components covered by various types of DLC coatings operating under dry, wet, or lubrication conditions were tested by some researchers [31,68,69,70,71,72,73].

Benedetti et al. [70] studied lubricated rolling-sliding contacts using disc-on-disc tester configuration for different material arrangements. The bronze-quenched and tempered steel was a reference couple. A steel-steel couple with various surface modifications including plasma nitriding, WC/C, WC-CrN, and DLC coatings were evaluated to ameliorate the lubricated wear behaviour of the tribo-pair. For all material conditions, limited surface modifications and visible performance enhancement with respect to the reference bronze-steel coupling occurred.

Tuszynski et al. [37] studied abrasive wear and scuffing on a pin- and-vee-block tribotester operating in a sliding mode. Pitting was investigated on a cone–three-ball tribotester operating in a rolling mode. The material of the studied specimens (pins, vee blocks, and cones) was 18CrNiMo7-6 case-hardened steel. Two types of DLC layers were evaluated, including the W-DLC one and the W-DLC/CrN one. The vee blocks and cones were coated. The contact zones studied were lubricated alternatively by two kinds of industrial gear oils, namely one case with a mineral and the other one with a synthetic PAO base, as pure oil or contaminated with solid particles from a coal mine. They found that the highest decreasing of abrasion, scuffing, and pitting of specimens made of 18CrNiMo7-6 steel was obtained using the W-DLC/CrN protective layer. Such a layer provided a good protection also under lubrication of contact zone by contaminated oil.

## 3. Milling Process

Milling is one of the machining methods that enable the execution of various planes and surfaces, recesses, keyways, threaded connections, and toothings. The main movement, which is a rotary movement, is performed by the tool: milling cutter/milling head. The feed movement (feed) during milling is most often performed by the processed element. It depends on the type of milling machine, and on its kinematic system. In milling, multiple cutting edges participate in the machining of the tool so that workpiece material in the form of chips is efficiently removed.

**Figure 2:**
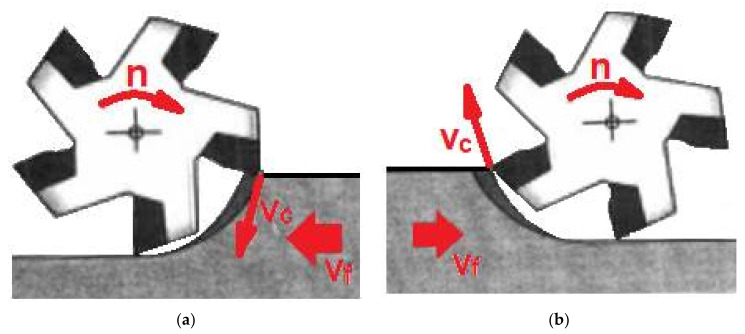
A schematic layout of climb milling and up milling. (**a**) climb type; (**b**) classical type.

There are two types of milling [74]:

- Climb—when the cutter performs a rotary motion in accordance with the movement of the workpiece feed. The result is an even and matt machined surface, allowing the weakening of cutter wear and prolonging its durability. For milling to be effective, minimal play in the drive mechanism is necessary. It is caused by the process of plunging the individual blades of the cutting tools into the workpiece under the conditions of generating the chip from high to low thickness (Figure 2). Most of the heat generated is transferred to the chip removed behind the cutter, weakening the occurrence of the chips’ re-cutting phenomenon. The values of the selected cutting parameters are much higher compared to up-cut milling. The milling process analyzed in the present study concerns the concurrent type.
- Conventional—when the cutter rotates in the opposite direction to the feed of the workpiece (Figure 2).

According to [75], the machining process is characterized by many parameters including cutting tool, machine tool, machining process and parameters, workpiece, cutting fluid, and application method. Also important are their interactions with each other influencing cutting forces and power, chip formation mechanism and morphology, residual stresses, workpiece-tool-chip interface temperature, etc. Additionally, the performance indicators including tool life/wear, surface integrity, accuracy, microstructure, part distortion, and stability depend on such interactions.

The milling process is often characterized by the three parameters including feed per tooth, depth of cut, and cutting speed. With the enhancement of feed and depth of cut, the cutting force components occurring during the milling process of the elements made of ductile iron (DI) and austempered ductile iron (ADI) also increased. Higher values of cutting force components were observed for ADI elements. Cutting force component values depended less on cutting speed during the milling process [76].

For the face milling process of elements made of Al alloy 7075 obtained via the SSM (semi solid metal) casting process, the best effect for the cutting force was obtained under a minimum level for cutting speed, feed per tooth, and depth of cut. On the contrary, for average arithmetic roughness, the optimal processing regime occurred for minimum values for cutting speed and feed per tooth, and for average values of cutting depth [77].

Ahmad et al. [78] noticed that cutting speed, radial depth of cut, and feed rate contribute the most to the rate of tool life during the milling process. During the machining of hard-to-machine materials such as titanium alloys, the increase of cutting speed can have an insignificant effect on the tool wear and resultant roughness of the machined surface [79] or can shorten the tools life even by 20% [80]. Surprisingly, Tian et al. [81] found that cutting forces firstly weakened and then enhanced with the increase of cutting speed during milling with Sialon ceramic tools, the elements made of Incontel 718.

### The End Milling Process of Bobbin Case

The end milling process was investigated by several researchers.

Azim et al. [82] studied the effect of milling process parameters on roughness parameters of injection mold made of mild steel grade 60 and machined using the CNC end milling machine (five-axis). The combination of parameters for the milling process including a spindle speed of 800 rpm, feed rate of 10 mm/rev, and depth of cut of 0.5 mm allowed minimum values of surface roughness parameters to be obtained. The spindle speed turned out to be the most crucial factor affecting the surface roughness of mild steel products, while the feed rate was insignificant.

Odedeyi et al. [83] investigated the tool wear propagation variations in the end milling of stainless steel 316 with inserts made of TiAlN-PVD-multi-layered coated carbides. They found that cutting speed significantly affected the machined flank wear values. With an enhance of cutting speed, the flank wear values weakened. Additionally, the significant flank wear turned out to be the most important failure mode affecting the tool life.

Vereş et al. [84] stated that the main performance factor of the end milling cutters is their durability. With the sound analysis of the tool engaged in the cutting process, they can forecast the future durability of the milling cutters. Alternatively, the future durability can be evaluated via the analysis of the chip behaviour, which can also be useful in the optimization of the cutting tools. The authors studied in detail the chip behaviour of 5-fluted end milling cutters with different performances and geometries when approaching several types of cutting operations of 42CrMo4 alloy steel.

Bhardwaj et al. [85] experimentally studied the effect of end milling operation of AISI 1019 steel using carbide inserts in surface roughness. They found that the cutting speed, feed, and nose radius had a significant effect on surface roughness.

Thien and Trung [86] experimentally studied the milling process of SCM440 steel with TiN coated cutting tool with a radius tip of 0.5 mm. They found that among the three parameters studied of cutting parameters such as cutting speed, feed rate, and cutting depth, the feed rate was the only parameter that significantly affects the cutting force.

Vereschaka et al. [87] calculated strains and deformations of composite mills, being combinations of a carbide cutting part and a steel shank and compared them with those for solid carbide mills. They also compared the accuracy parameters of machining with monolithic mills and two-component mills with various shank materials. After conducting cutting tests in milling Al alloy with monolithic and two-component end mills with multilayer composite nano-structured coatings, they found that the use of such coatings can weaken strains and deformations, allowing the accuracy of machining to be improved.

Chen et al. [88] proposed an abductor induction mechanism (AIM) polynomial network, with material properties as input for the prediction of tool geometry. They calibrated the model using the following materials: NAK80, quenched SKD61, annealed SKD61, quenched S45C, annealed S45C, SUS316L, SUS304, and Ti6Al4V.

Biriş and Racz [89] studied burrs dimensions decrease that appears after the milling process together with an approach to limit the burrs resulting after this process. To limit burrs dimensions, the milling process was executed with various cutting parameters and strategies.

According to Malekan et al. [90], tool wear is affected by cutting parameters and conditions, tool geometry and materials (coating and base materials), and the workpiece material. The authors compared the performance of three tools for high-speed end milling while cutting the stainless steel. The tools were made of WC-Co, with various geometrical parameters and coatings (TiAlN and AlCrN). They found the flank wear as the most intensive damage mechanism at the cutting edge. Stresses and degree of wear at the latter were dictated by the radial depth of cut and feed per tooth.

The analyzed milling process of the bobbin case has been conducted using the Haas three-axis CNC milling machine, model VF4.

## 4. Materials and Methods

### 4.1. Bobbin Case

**Figure 3:**
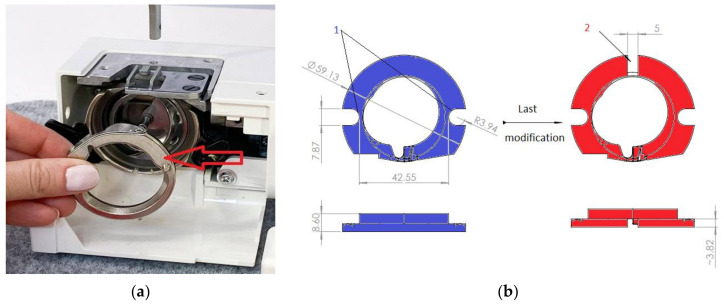
Bobbing case analyzed. (**a**) A view of the original bobbin case; (**b**) Two grooves 1 made during earlier modifications and the one groove 2 made after last modification of the bobbin case (units in mm).

The subject of the analysis was a milling chuck that fastens the bobbin case to perform the necessary milling operations in it. The said bobbin case was supplied directly from the manufacturer, but its use in a lockstitch specialized for a specific sewing process required modification of the shape of the bobbin case. The modification consisted in making a milling (shallow groove) perpendicular to the common symmetry plane of such grooves made in the original bobbin case (Figure 3) during its previous modifications. These grooves were also used to fasten the bobbin case in the analyzed milling chuck and to place a series of bobbin cases on the wire guides transporting them to this chuck. The width of said milling was 5mm and the depth was about 4mm.

The milling of the mentioned type was performed in the production process with the use of a three-axis numerically controlled milling machine (CNC) (Haas Vorster, Zduńska Wola, Poland). 

### 4.2. Scheme of the Milling Chucks

**Figure 4:**
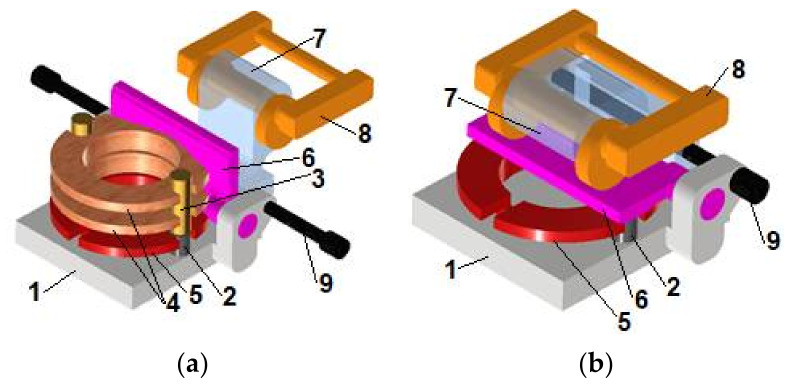
Scheme of milling chuck. (**a**) In open state—during supply of the bobbin case to the milling chuck; (**b**) In close state—directly before milling the groove in the bobbin case. 1—milling check support, 2—locating pin, 3—wire guides, 4—series of bobbin case delivered from a supply bin on the production line (not presented here), 5—machined bobbin case, 6—swivel arm, 7—lever, 8—eccentric, and 9—locking pin.

Before the milling, the series 4 of bobbin cases are placed in the set of two parallel wire guides 3 (Figure 4a) with diameters close to the width of grooves 1 (Figure 3b). The distance between the axes of such guides 3 related to the distance between centres of semi-cylindrical ends of the mentioned groves 1 (Figure 3b).

Supplying through such profiled wire guides 3 enabled the bobbin case 4 to fall in a specific position in relation to the milling chuck 1, and more clearly, to its locating pins 2. The bobbin case 5 was pressed against the supporting surface of the chuck support 1 by means of a swivel arm 6, the bottom plane of which acted on one of the front planes of the bobbin case 5 (Figure 4b). The swivel arm 6 was pivotally mounted relative to the milling chuck support 1, and in the open state reached vertical position (Figure 4a). In the close state, the upper surface of such an arm 6 was pressed against an eccentric 8 pivotally mounted relative to the angled lever 7 (Figure 4b). Such a lever 7 was also pivotally mounted relative to the milling chuck support 1, and in the open state reached the position shown in Figure 4a. The rotation of the eccentric 8 by a given angle was performed manually.

To allow a proper action of eccentric 8, the angled lever 7 was blocked in a specific position relative to the milling chuck support 1 by means of the two locking pins 9 (Figure 4b). The action of the eccentric 8 generated a force pressing the swivel arm 6 against the upper front plane of the machined bobbin case 5, and then, pressing of the bottom front plane of the bobbin case 5 against the supporting surface of the milling chuck support 1. This allowed generation of frictional forces in the contacts of the bobbin case 5 with the supporting surface of the milling chuck support 1 and with the bottom plane of the swivel arm 7. The distribution of these forces prevented the bobbin case 5 from moving during milling under the influence of the cutting forces caused by the movement coming from the cutting tool, i.e., the end mill.

### 4.3. Loading of the Milling Chuck

#### 4.3.1. Assumptions for the Milling Process

It was assumed that:

- Milling was performed with a rough three-leaf end milling cutter Ø4 made of HSS and a four-leaf end milling cutter Ø5 with inserts made of cemented carbide.
- The milling process was conducted with appropriately selected milling parameters presented in Table 1.
- Maximally three teeth of the cutting tool in contact with the side walls of the machined groove were loaded during milling.
- The workpiece was an isotropic flexible body made of 40H/1.7053 structural alloy steel for quenching and tempering (QT) (PN-EN 100083) with the Yield stress $R_{e}$ equal to 780 MPa.
- Approximate surface roughness parameter Ra reached values of 3.2 μm.

#### 4.3.2. The Derivative Parameters of the Milling Process

***Feed per tooth (*** $f_{z}$ ***)* [*mm/t*]**

The feed per tooth $f_{z}$ of the milling cutter was calculated from Equation (1) [91].

(1) $$f_{z}=\frac{V_{f}}{n\cdot ZEFF}$$

where:

$V_{f}$—cutting speed [mm/min];

n—rotational speed [rpm];

ZEFF=3—number of teeth in the active field, as assumed.

The determined values of $f_{z}$ for the two cutters used are presented in Table 2.

***Specific cutting resistance*** $k_{c}$ **[*N/mm^2^*]**

The specific cutting resistance $k_{c}$ during end milling was defined by Equation (2) [91].

(2) $$k_{c}=\frac{F_{c}}{A_{D}}$$

where: 

$F_{c}—$ peripheral force component [N];

$A_{D}—$ cutting area [mm^2^].

***Peripheral component*** $F_{c}$ **[*N*]**

Rearranging Equation (2) [91], the circumferential force component $F_{c}$ during end milling was calculated from Equation (3) [91].

(3) $$F_{c}=k_{c}\cdot A_{D}$$

***Cutting area*** $A_{D}$ **[*mm^2^*]**

The cutting area $A_{D}$ was calculated from Equation (4) [91].

(4) $$A_{D}=b\cdot h$$

where: 

b=0.5 [mm]—cutting depth;

$h\approx f_{z}$ (chip thickness = feed per tooth) [mm].

The determined values of cutting area $A_{D}$ for the two used cutters are presented in Table 3.

The specific cutting resistance $k_{c}$ was calculated from Equation (5) [91].

(5) $$k_{c}=k_{c1.1}\cdot h^{y_{c}-1}$$

where: 

$k_{c1.1}=1500$—factor with assumed value;

$y_{c}=0.72$—exponent with assumed value.

The determined values of $k_{c}$ and $F_{c}$ for the two cutters used are shown in Table 4.

#### 4.3.3. Model of Loading of the Bobbin Case before and during Milling

It was assumed that the analyzed milling process was conducted under dry conditions. Before the milling process, the bobbin case is loaded from the action of the swivel arm loaded by the formal force $F_{n}$. During the milling process, the bobbin case is additionally loaded by the torque from the actions of the cutter blades.

**Figure 5:**
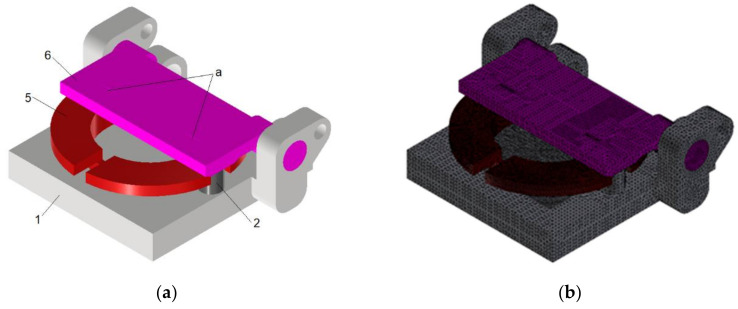

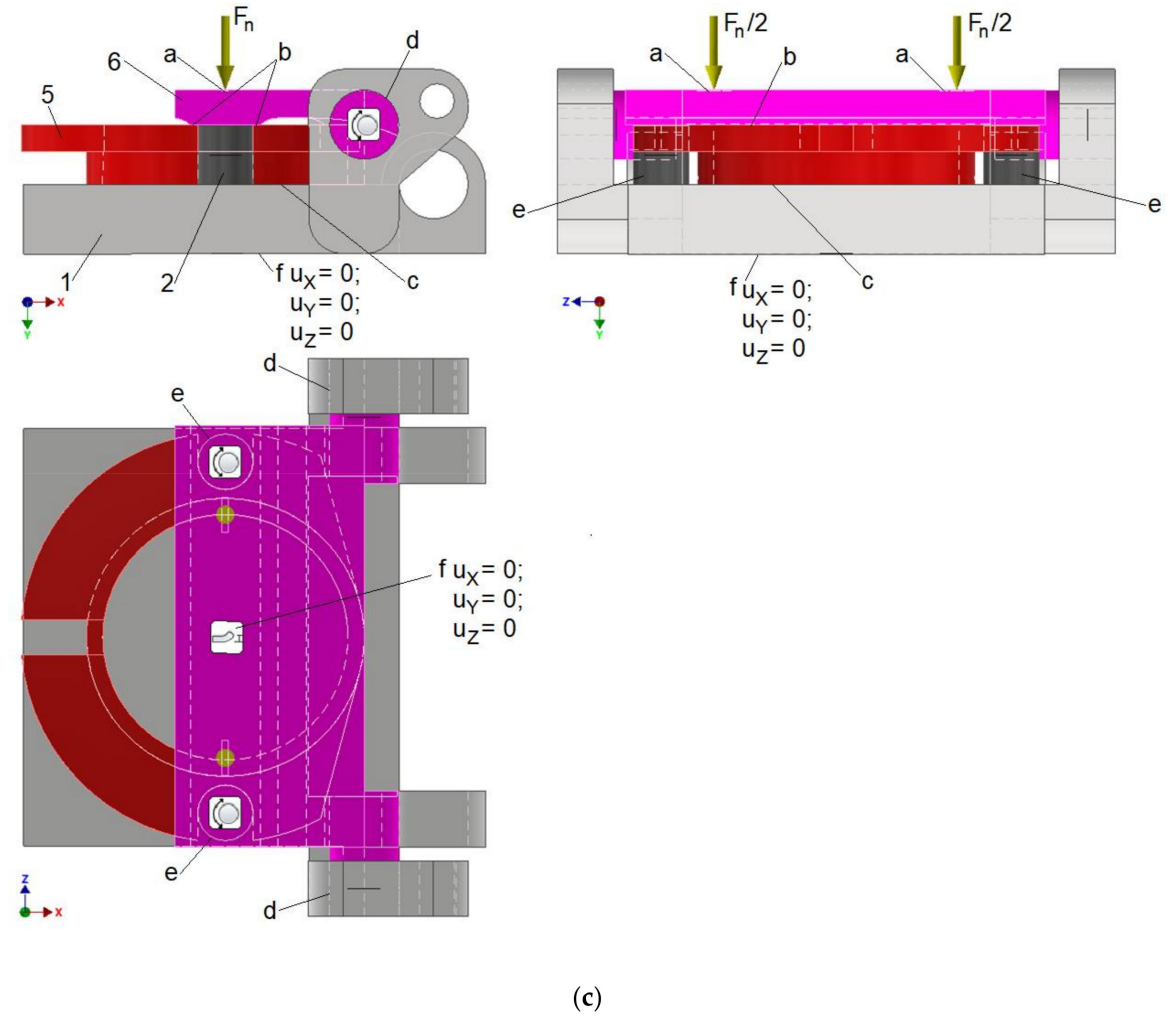
The model for determining contact pressure between eccentric and swivel arm. (**a**) Geometry of components; (**b**) Finite element grid; and (**c**) Boundary conditions.

The model of the bobbin case positioned in the milling chuck before the milling process and machined during the end milling process was elaborated using the FEM. The model comprised the body of the bobbin case 5 with the shape and dimensions close to the real ones, the milling chuck support 1 with two locating pins 2 fixed to it, and the swivel arm 6 (Figure 5 related to Figure 4). The latter was loaded by two equal components of normal force $F_{n}$ applied in the form of equivalent normal pressures to the lower surfaces of two identical, symmetrically spaced cuboidal grooves ‘a’ (Figure 5a) of shallow depth and lengths equal to those component cams of the eccentric 8 (Figure 4). These grooves partially prevented the generation of unnecessary unrealistic stress concentrations. The position of these grooves correlated with this of the two contact zones between the eccentric 8 and the swivel arm 6 (Figure 4). This correlation occurred in such a way that the center of the contact zone coincided with this of the bottom surface of the groove. Additionally, through this center the mentioned component of normal force $F_{n}$ passed (Figure 5). The isotropic material model of such parts comprised properties of stainless steel with the Young modulus equal to 210,000 MPa and Poisson number equal to 0.3. The grid of tetrahedral finite elements was generated in Autodesk Inventor v.2021 software. Each finite element comprised four nodes, each of which possessing three degrees of freedom being displacement along the axes *X*, *Y*, and *Z* of the Cartesian coordinate system. Such displacements were marked by $u_{X}$, $u_{Y}$, and $u_{Z}$, respectively. The 3D triangle contact zones with 3 nodes correlated to the relative nodes of neighbouring tetrahedral finite elements were generated in the interfaces (Figure 5c):

- ‘c’ between the bottom front plane of the bobbin case 5 and the upper surface of the milling chuck support 1;
- ‘d’ between cylindrical surfaces of two pins of the swivel arm 6 and related to them two holes in the milling chuck support 1;
- ‘e’ between the upper front plane of the bobbin case 5 and the bottom plane of the swivel arm 6.

The friction sliding was allowed between contact and target surfaces with friction coefficient of 0.3 providing numerical stability of solution. The steady state analysis was conducted, and the nonlinear algorithm was applied due to nonlinear character of contact element behaviour. 

The cutting torque from cutter blades was introduced by tangential pressure placed anti-symmetrically on two treated parallel surfaces of the bobbing case 5. The values of this tangential pressure were determined from Equation (6).

(6) $$p_{t}=\frac{F_{c}}{A_{t}}$$

where: 

$A_{t}=45.2\;{\mathrm{mm}}^{2}$—area of the treated surface of the bobbin case 5. 

The determined values of the tangential pressure $p_{t}$ for the two cutters used are shown in Table 5.

The study on the effect of average finite element size on the solution convergence process was conducted. The criterion was the stabilizing of the maximum values contact pressure in the contact zone between the swivel arm 6 and bobbin case 5 with the decrease of the average finite element size. 

#### 4.3.4. Model of Loading of the Swivel Arm

To counteract the cutting torque generated by the cutter blades in contact with the machined bobbin case 5 during a milling process, the bobbin case 5 should be fixed relative to the milling chunk (Figure 4). It should be realized by the frictional torques generated in the contact zones between the swivel arm 6 and a bobbin case 5 and between the bobbin case 5 and milling check support 1 instead of by shape interactions in the contact zones between the bobbin case and locating pins 2 (Figure 4). The latter serve to ensure the initial correct positioning of the bobbin case 5 relative to the support 1. The generation of the abovementioned friction torques requires prior generation of the appropriate normal force $F_{n}$ followed by a resulted contact pressure $p_{c5-6}$ between the bobbin case 5 and the swivel arm 6 and consistent contact pressure $p_{c1-5}$ between the bobbing case 5 and the support 1. Such contact pressures arise because of the interaction of the shaped surfaces of the eccentric 8 with the upper surface of the swivel arm 6.

The necessary value of normal force $F_{n}$ is obtained from Equation (7).

(7) $$F_{n}=\frac{F_{c}\cdot d_{c}}{\mu \cdot 0.5\cdot \left(B_{aver1-5}+D_{aver5-6}\right)}$$

where: 

$2\cdot A_{1-5}=122\;{\mathrm{mm}}^{2}$—area of two contact zones between the bobbin case 5 and the swivel arm 6;

$B_{aver1-5}=39\;\mathrm{mm}$—distance between centres of contact zones between the bobbin case 5 and the swivel arm 6;

$A_{5-6}=275\;{\mathrm{mm}}^{2}$—area of a ring contact zone between the bobbin case 5 and milling check support 1;

$D_{aver5-6}=37\;\mathrm{mm}$—average diameter of a ring contact zone between the bobbin case 5 and the milling check support 1;

μ=0.3—the friction coefficient in dry contact zones between the bobbin case 5 and the swivel arm 6 and between the bobbin case 5 and the milling check support 1.

The determined values of the normal force $F_{n}$ for the two cutters used are shown in Table 6.

**Figure 6:**
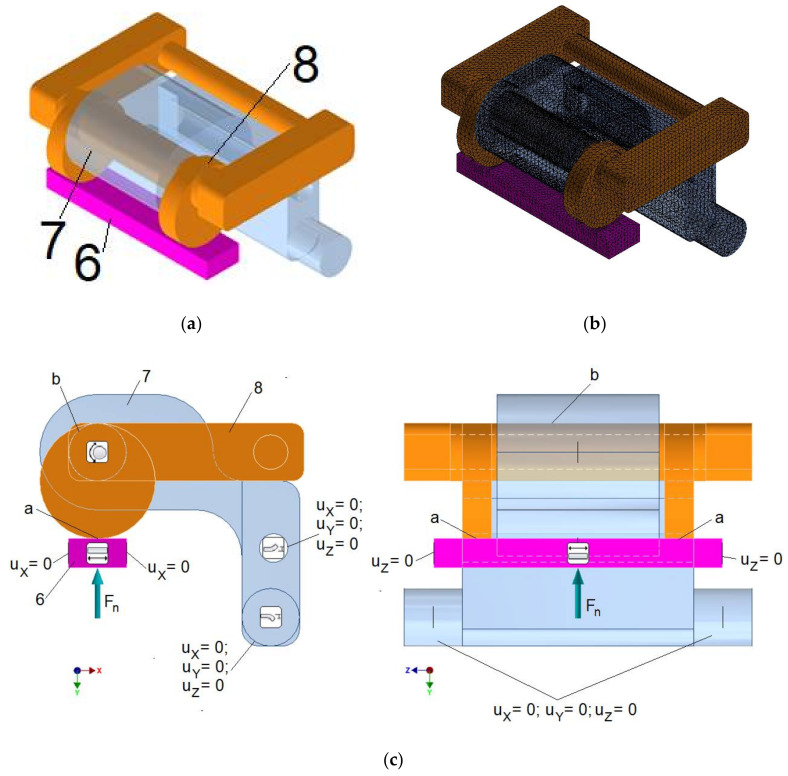
The model for determining contact pressure between eccentric and swivel arm. (**a**) Geometry of components; (**b**) Finite element grid; (**c**) Boundary conditions.

The model for determining contact pressure in two contact zones between eccentric 8 and swivel arm 6 (Figure 6 relative to Figure 4) was presented in Figure 6. This model comprised a segment of the swivel arm 6, lever 7, and the eccentric 8, the geometry of which is shown in Figure 6a. The isotropic material model of such parts comprised properties of stainless steel with the Young modulus equal to 210,000 MPa and Poisson number equal to 0.3. The grid of tetrahedral finite elements is presented in Figure 6b. Each finite element possessed four nodes with three degrees of freedom, namely, displacements $u_{X}$, $u_{Y}$, and $u_{Z}$, along the axes *X*, *Y*, and *Z*, respectively. In the interface ‘b’ (Figure 6c) between the cylindrical surface of the eccentric 8 and the upper hole in the lever 7 and in two interfaces ‘a’ (Figure 6c) between the cylindrical cam surfaces of eccentric 8 and plane of segment of the swivel arm 6, the 3D triangle contact zones were generated, with 3 nodes correlated to the relative nodes of neighbouring tetrahedral finite elements. The friction sliding was allowed in interfaces ‘a’ and ‘b’ with friction coefficient of 0.3 providing numerical stability of solution. The cylindrical surfaces of two bottom pins of the lever 7 and its bottom hole was fixed (Figure 6c). The bottom surface of the segment of the swivel arm 6 was loaded by normal force $F_{n}$ and its displacement along the axis Y was allowed (Figure 6c). The steady state analysis was conducted, and the nonlinear algorithm was applied due to nonlinear character of contact element behaviour. The effect of average size of finite element on the solution convergence was established with the criterion of the maximum value of normal contact pressure. 

The distance D between interfaces ‘a’ on the swivel arm 6 and the axis of the eccentric 8 was estimated from Equation (8).

(8) $$D\approx \frac{D_{ecc}+d_{axis}}{2}-\delta_{total}$$

where:

$D_{ecc}=30\;\mathrm{mm}$—diameter of the cylindrical cam of the eccentric 8;

$d_{axis}=15\;\mathrm{mm}$—diameter of the axis of eccentric 8 mating with the hole in the lever 7 (interface ‘b’);

$\delta_{total}$—the maximal vertical distance between the most bottom points of unloaded cylindrical cam of the eccentric 8 when the position of lever 7 is fixed for milling operation, and the initial position of unloaded interfaces ‘a’ of the swivel arm 6 [mm]. 

It was assumed that the maximal vertical distance $\delta_{total}$ was below twice the value of total indentation depth δ, corresponding to the occurrence of average values of contact pressure $p_{aver}=\frac{F}{2\cdot a_{ecc}\cdot h_{ecc}}$ [92] in the interference ‘a’ under loading by force F, and it was given by Equation (9) [92].

(9) $$\delta_{total}\approx 2\cdot \delta =2\cdot 2\cdot \frac{F}{h_{ecc}}\cdot \frac{1-\nu^{2}}{\pi \cdot E}\cdot \left[1+ln\left(\frac{h_{ecc}^{2}}{\frac{1-\nu^{2}}{\pi \cdot E}\cdot \frac{F}{h_{ecc}}\cdot D_{ecc}}\right)\right]$$

where:

ν=0.3—Poisson ratio of steel;

E=21,0000 MPa—Young modulus of steel;

$h_{ecc}$—the thickness of the cylindrical cam of the eccentric 8;

$a_{ecc}=2\cdot \sqrt{\frac{D_{ecc}}{\pi }\cdot \frac{F}{h_{ecc}}\cdot \frac{1-\nu^{2}}{E}}$—the half width of contact zone between cylindrical cam of eccentric 8 and the interface ‘a’ on the swivel arm 6 under loading by force F [92]. 

### 4.4. Model of Wear in Contact Zones between an Eccentric a Swivel Arm

**Figure 7:**
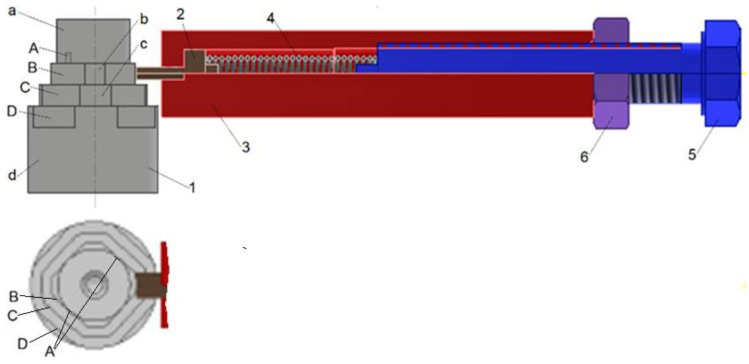
The model of wear in contact zones between an eccentric and a swivel arm. 1—shaft, 2—punger, 3—support, 4, spring, 5—set screw, 6—lock nut.

To determine wear in contact zones between an eccentric cam and a swivel arm, the physical model was elaborated and is presented in Figure 7.

The model comprised the shaft 1 with four shaft shoulders. On the consecutive shoulder, the following cuts at the length of 5 mm were made, from the side of their successive faces, symmetrically distributed with respect to the shaft axis:

- on the shoulder a with the smallest diameter of 17.5 mm, two cuts A were made with a wall distance of 17 mm;
- on the shoulder b with the diameter of 20 mm, four cuts B were made with a wall distance of 17 mm;
- on the shoulder c with the diameter of 25 mm, four cuts C were made with a wall distance of 22 mm;
- on the shoulder d with the diameter of 30 mm, four cuts D were made with a wall distance of 27 mm.

In the regions of cuts at their lengths of 5 mm, the cylindrical surfaces of an individual shaft shoulder mated with the front surfaces of movable plunger 2, in which the cylindrical surface was joined with the cylindrical hole of the support 3. The plunger 2 can make reciprocal displacement limited from one side by the bottom front plane of the hole and from the other by the one front plane of the compression spring 4. The second front plane of the spring 4 mated with the front plane of the set screw 5. The position of the set screw 5 relative to the support 3 was fixed by the lock nut 6.

The parameters of the compression spring 4 are presented in Table 7. The spring rate R was determined from Equation (10).

(10) $$R=\frac{G\cdot d}{8\cdot n}\cdot {\left(\frac{d}{D}\right)}^{3},$$

where: 

G—the modulus of elasticity for steel—material used for the spring wire;

d—wire diameter;

n—the number of active spring coils;

$D=0.5\cdot \left(D_{e}+D_{i}\right)$—the average diameter of spring;

$D_{e}$—external diameter of spring;

$D_{i}$—internal diameter of spring.

If the one front plane of spring 5 (the left one in Figure 7) was fixed, the spring deflection was measured as the displacement of the right front spring plane from the position related to the free spring length to the position determined by the position of the front plane of the set screw 5 (Figure 7). The initial operational spring deflection varied from the minimal value $f_{min}^{init}$ to its maximum value $f_{max}^{init}$ (Table 8). The forces related to those spring deflection values were $F_{min}^{init}$ and $F_{max}^{init}$, respectively, and were determined from Equation (11).

(11) $$F=\left\{\begin{array}{c} F_{min}^{init}=R\cdot f_{min}^{init}\\ F_{max}^{init}=R\cdot f_{max}^{init} \end{array}\right.,$$

The initial average contact pressure between the front plane of plunger 2 (Figure 7) and the cylindrical surface of the rotating shaft shoulder was determined from Equation (12).

(12) $$p_{c}=\frac{F}{2\cdot b\cdot h_{ecc}}=\frac{F}{2\cdot h_{ecc}\cdot \sqrt{\frac{8\cdot F\cdot R_{x}}{\pi \cdot h_{ecc}}\cdot \frac{1-\nu^{2}}{E}}}$$

where: 

$h_{ecc}=3.2\;\mathrm{mm}$—the width of the fin of the plunger 2 (Figure 7);

$R_{x}=\frac{D_{x}}{2}\;\left[\mathrm{mm}\right]$—the radius of the cylindrical surface of the shoulder of the shaft 1 (Figure 7) with diameter $D_{x},\;\mathrm{where}\;x=a,\;b,\;c,\;\mathrm{or}\;d$;

E=210,000 MPa—the Young’s modulus of steel;

ν=0.3—the Poisson number of steel;

$2b=2\sqrt{\frac{8\cdot F\cdot R_{x}}{\pi \cdot h_{ecc}}\cdot \frac{1-\nu^{2}}{E}}$—the width of contact zone between the elastic cylinder of the radius $R_{x}$ and of the length $h_{ecc}$ and the elastic half-space.

During the experiments, the wear took place in the contact zone. The average contact pressure varied according to the Equation (13).

(13) $$p_{c}=\frac{F}{h_{ecc}\cdot R_{x}\cdot \theta }=\frac{F}{2\cdot R_{x}\cdot 2\cdot \arccos\left(\frac{R_{x}-h}{R_{x}}\right)},$$

where: 

h—the recess of the shoulder surface of the rotating shaft into the plunger fin (measured from the front plane of the fin).

During the experiment, the consecutive series involving 50 shaft rotations with the speed equal to 18 rpm was realized. During the first series the shaft turned right, during the next one the shaft turned left, and so on, up to reaching:

- 1000 rotations of shaft under load by the force $F_{max}$ for the shaft shoulder with the lowest diameter;
- 500 rotations of shaft under load by the force $F_{min}$ for the other three shaft shoulders.

The test stand for determining the wear in contact zone between the plunger fin 2 and the cylindrical surface of the shaft shoulder 1 (Figure 7) was realized using a universal lathe equipped with a three-jaw chuck for securing the shaft 1, which on the other side was supported by its center in the rotary joint of the lathe tailstock. The support 3 with the movable plunger 2, the spring 4, the set screw 5, and the lock nut 6 were mounted in the lathe holder. In the start of each series, the lathe holder was placed in such a way that the vertical front plane of the plunger fin was parallel to the axis of the shaft and:

- tangent to the wall of the cut A made in the shaft shoulder with the smallest diameter;
- at 1 mm from the wall of the cut B, C, or D made in the other respective shaft shoulder.

This resulted in the spring deflections and respective forces loading the contact zone between the movable plunger 2 and the shaft shoulder 1 (Figure 7). Values of such spring deflections and forces are shown in Table 8.

**Figure 8:**
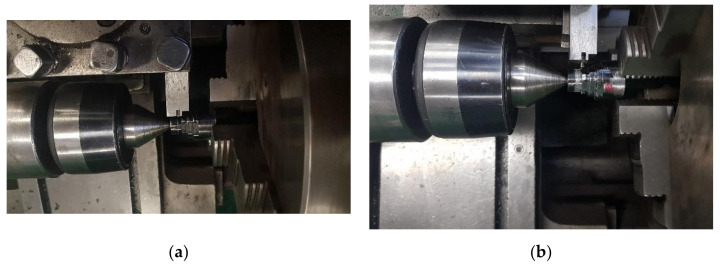
The research stand for determining the wear in contact zone between the plunger fin and the cylindrical surface of the shaft shoulder. (**a**) For the shaft shoulder with the lowest diameter equal to 17.5 mm and two cuts A; (**b**) for the shaft shoulder with the diameter equal to 20 and four cuts B.

Two views of the test stand are shown in Figure 8a for the shaft shoulder with the diameter equal to 17.5 mm and two cuts A, and in Figure 8b for the one with the diameter equal to 20 and four cuts B, respectively.

The wear can be determined using the Archard law described by Equation (14), by Ejtehadi et al. [93], as cited in [94].

(14) $$V_{w}=k_{A}\cdot F_{n}\cdot s_{w},$$

where:

$V_{w}$—the worn volume [mm^3^];

$F_{n}$—the normal applied load [N];

$s_{w}$—the sliding distance [mm];

$k_{A}$—the wear coefficient [mm^2^N^−1^].

Watrin et al. [94] proposed a related Equation (15) between the wear coefficient $k_{A}$ and the sliding distance $L_{w}$.

(15) $$k_{A}=6\cdot 10^{-7}\cdot L_{w}^{-0.58},$$

In the present study, it was assumed that the linear wear intensity $I_{hi}$ for both the plunger fin and the mating shaft shoulder can be calculated using Equation (16).

(16) $$I_{\mathrm{hn}}=k_{n}\cdot p_{a}^{2};\;n=1,\;2,$$

where: 

*k*_1_ and *k*_2_—the wear intensity factor for the plunger fin and the shaft shoulder, respectively [Pa^−2^];

$p_{a}$—averaged pressure in contact zone between the plunger fin and the shaft shoulder [Pa].

Moreover, it was assumed that the linear wear intensity may depend on the slip velocity, and the function describing this relationship has the form described by Equation (17). When selecting the form of the function $f\left(v\right)$, it was considered that for the speed v=0, the function $f\left(v\right)$ must also reach zero value.

(17) $$I_{\mathrm{hn}}=k_{\mathrm{an}}\cdot f\left(v\right)\cdot p_{a}^{2}=k_{\mathrm{an}}\cdot \left(A_{v}\cdot v^{2}+B_{v}\cdot v\right)\cdot p_{a}^{2};\;n=1,\;2,$$

where:

$k_{\mathrm{an}}$—the modified wear intensity factor for the plunger fin and the shaft shoulder, respectively [Pa^−2^];

$A_{v}$—the first factor of the function $f\left(v\right)$ [m^−2^s^2^];

$B_{v}$—the second factor of the function $f\left(v\right)$ [m^−1^s].

The wear intensity factor $k_{i}$ characterizing the linear wear intensity $I_{\mathrm{hn}}$ was estimated from from Equation (18).

(18) $$W_{n}=w_{z}\cdot t_{\mathrm{corn}}=I_{\mathrm{hn}}\cdot S\cdot \sum_{j=1}^{j=M}v_{b}\left(j\right)\cdot t_{u}\left(j\right)\;\Rightarrow k_{n}=\frac{W_{n}}{R_{b}\cdot p_{a}\cdot \sum_{j=1}^{j=M}v_{b}\left(j\right)\cdot t_{u}\left(j\right)};\;n\;=1,\;2,$$

where:

$w_{z}=I_{\mathrm{hn}}\cdot S\cdot v_{b}^{\mathrm{aver}}$—volumetric wear rate [m^3^⋅s^−1^];

$v_{b}^{\mathrm{aver}}=\frac{l_{z}}{t_{\mathrm{corn}}}$—average speed of the shaft shoulder cylindrical surface relative to the plunger fin during all time of the shaft rotation [ms^−1^];

$t_{\mathrm{corn}}$—summary time of shaft shoulder rotation series [s];

$l_{z}=v_{b}^{\mathrm{aver}}\cdot t_{\mathrm{corn}}=\sum_{j=1}^{j=M}v_{b}\left(j\right)\cdot t_{u}\left(j\right)$—wear distance [m]; 

$W_{n}$—volumetric wear of the plunger fin (n = 1) or the shaft shoulder (n = 2) [m^3^];

$S=R_{b}/p_{a}$ contact area between the plunger fin and the mating shaft shoulder [m^2^];

$R_{b}$—reaction in the contact zone between the plunger fin and the mating shaft shoulder [N].

Considering Equations (16)–(18), the dependence between the factor $k_{n}$ and the modified factor $k_{\mathrm{an}}$ given by Equation (15) was obtained.

(19) $$k_{n}=k_{\mathrm{an}}\cdot \left(A_{v}\cdot v^{2}+B_{v}\cdot v\right);\;n\;=1,\;2$$

The mass wear $M_{2i}$ of the i-th plunger fin was determined as the difference of the new plunger mass $m_{i}^{new}$ and that of the worn one $m_{i}^{worn}$. The masses of the previously cleaned plungers were measured using a precise laboratory balance with an accuracy of 0.05 g. The corresponding volumetric wear $W_{2i}$ of i-th plunger fin was obtained from Equation (20).

(20) $$W_{2i}=\frac{M_{2i}}{\rho }=\frac{m_{i}^{new}-m_{i}^{worn}}{\rho };\;i=1,\;2,\;3,4,$$

where:

$M_{2i}$—the mass wear of the i-th plunger fin [kg];

$m_{i}^{new}$—mass of the new i-th plunger [kg];

$m_{i}^{worn}$—mass of the worn i-th plunger [kg];

$\rho =7800\;\mathrm{kg}/m^{3}$—density of steel.

The volumetric wear $W_{1i}$ of the i-th shaft shoulder mating with a front plane of the i-th plunger fin was determined using measurement of the *P* profile on the unit for roughness measurement. The obtained profiles were registered and the position of a front plane of the i-th plunger fin was superimposed on the plot of the recorded i-th *P* profile. The axial position of the i-th front plane corresponded to the operational position of the i-th plunger fin relative to the corresponding i-th shaft shoulder during their friction mating. In the radial direction, the assumed position of the i-th front plane was tangent to the nearest maximum height of the registered i-th *P* profile. The area between the assumed position of the top plane of i-th plunger fin and the i-th *P* profile line recorded at a length corresponding to the i-th plunger fin thickness of 3.2 mm corresponded to the cross-sectional area $A_{i}$ of the removed material of the i-th shaft shoulder. Measurements of areas $A_{i}$ for each of the three consecutive shaft shoulder with the largest diameters $d_{i}$ were made in four places. Such places were in two planes perpendicular to each other and rotated by an angle of 45 degrees to the planes of the cuts made on individual i-th shaft shoulder and pairs separated by value $s_{i}$. For the smallest shaft shoulder, only two measurements were made in a plane parallel to the planes of the two existing cuts on the shaft shoulder. The values from the four (two) relative measurement of areas $A_{i}$ were averaged and used to determine the volumetric wear $W_{i}$ of the corresponding i-th shaft shoulder from Equation (21) for three consecutive shaft shoulder with the largest diameters and from the Equation (22) for the shaft shoulder with the lowest diameter.

(21) $$W_{1i}=\frac{2\cdot \pi -4\cdot 2\cdot acos\left(\frac{s_{i}}{d_{i}}\right)}{2\cdot \pi }\cdot \pi \cdot d_{i}\cdot A_{iaver};i=1,\;2,\;3,$$

where:

$s_{i}$—separation between cuts made on the i-th shaft shoulder [m]; 

$d_{i}$—diameter of the cylindrical surface of the i-th shaft shoulder [m];

$A_{iaver}=0.25\sum_{j=1}^{j=4}A_{ij};i=1,\;2,\;3$—average value of the four cross-sectional area $A_{i}$ of the removed material of the i-th shaft shoulder.

(22) $$W_{1i}=\frac{2\cdot \pi -2\cdot 2\cdot acos\left(\frac{s_{i}}{d_{i}}\right)}{2\cdot \pi }\cdot \pi \cdot d_{i}\cdot A_{iaver},$$

where:

$A_{4aver}=0.5\sum_{j=1}^{j=2}A_{4j}$—average value of the two cross-sectional area $A_{4}$ of the removed material of the i-th shaft shoulder.

### 4.5. The Number of Cycles of Opening and Closing of the Milling Chunk until Reaching the Limit Value of the Force Pressing the Bobbin Case during Its Milling

Based on the obtained wear intensity factors for DLC coated steel of both the shaft shoulder and the steel plunger, the number of cycles of opening and closing the milling chunk can be estimated until decrease of the maximal vertical distance $\delta_{total}$ to its half initial value due to wear process of mating surfaces of eccentric and swivel arm. The latter value corresponds to the decrease of the force pressing the bobbin case to the value which cannot prevent micro-displacements of the bobbin case relative to the milling chunk. 

It was assumed that the change $\Delta \delta_{total\left(m\right)}$ of the maximal vertical distance $\delta_{total}$ during m-th cycle can be estimated from Equation (23).

(23) $$\Delta \delta_{total\left(m\right)}=\frac{{\left(0.5\cdot F\right)}^{2}\left[k_{1}\left(v_{ref}\right)+k_{2}\left(v_{ref}\right)\right]}{acos\left(\frac{0.5\cdot d_{axis}-\delta_{total}+\Delta \delta_{total\left(m\right)}}{0.5d_{axis}}\right)\cdot h_{ecc}};m=1,2,\ldots ,M,$$

where:

M—number of cycles until the maximal vertical distance $\delta_{total}$ reaches its half initial value due to wear process of mating surfaces of the eccentric cylindrical cams and the swivel arm;

0.5·F—force loading the single cylindrical cam of the eccentric; 

$d_{axis}=0.015$ m—diameter of the axis of the eccentric; 

$D_{ecc\left(m\right)}$—diameter of the eccentric cylindrical cam for the m-th cycle. For m=1 the $D_{ecc\left(1\right)}=0.030\;m$;

$\Delta \delta_{total\left(m-1\right)}$—change of the maximal vertical distance $\delta_{total}\left(m\right)$ for the (m-1)-th cycle. For the m=1 its value is of $\Delta \delta_{total\left(0\right)}=0$.

The wear intensity factors $k_{1}\left(v_{ref}\right),\;k_{2}\left(v_{ref}\right)$ were determined for the estimated average value sliding speed $v_{ref}=0.5\cdot D_{ecc\left(1\right)}\cdot \omega_{aver}$ of cylindrical cam of the eccentric relative to the mating surface of the swivel arm. An estimated average value of the angular speed $\omega_{aver}$ was equal to about 0.5π rad/s, which corresponds to the average value of sliding speed $v_{ref}$ equal to 0.24 m/s.

## 5. Results and Discussion

This section contains results of loading of the bobbin case and of swivel arm. The results of wear of elements of the model of the eccentric cam mating with the swivel arm are also presented. Additionally, the calculated number of cycles of opening and closing of the milling chunk until reaching the limit value of the force pressing the bobbin case during its milling was estimated.

### 5.1. Results of Loading of the Bobbin Case

**Figure 9:**
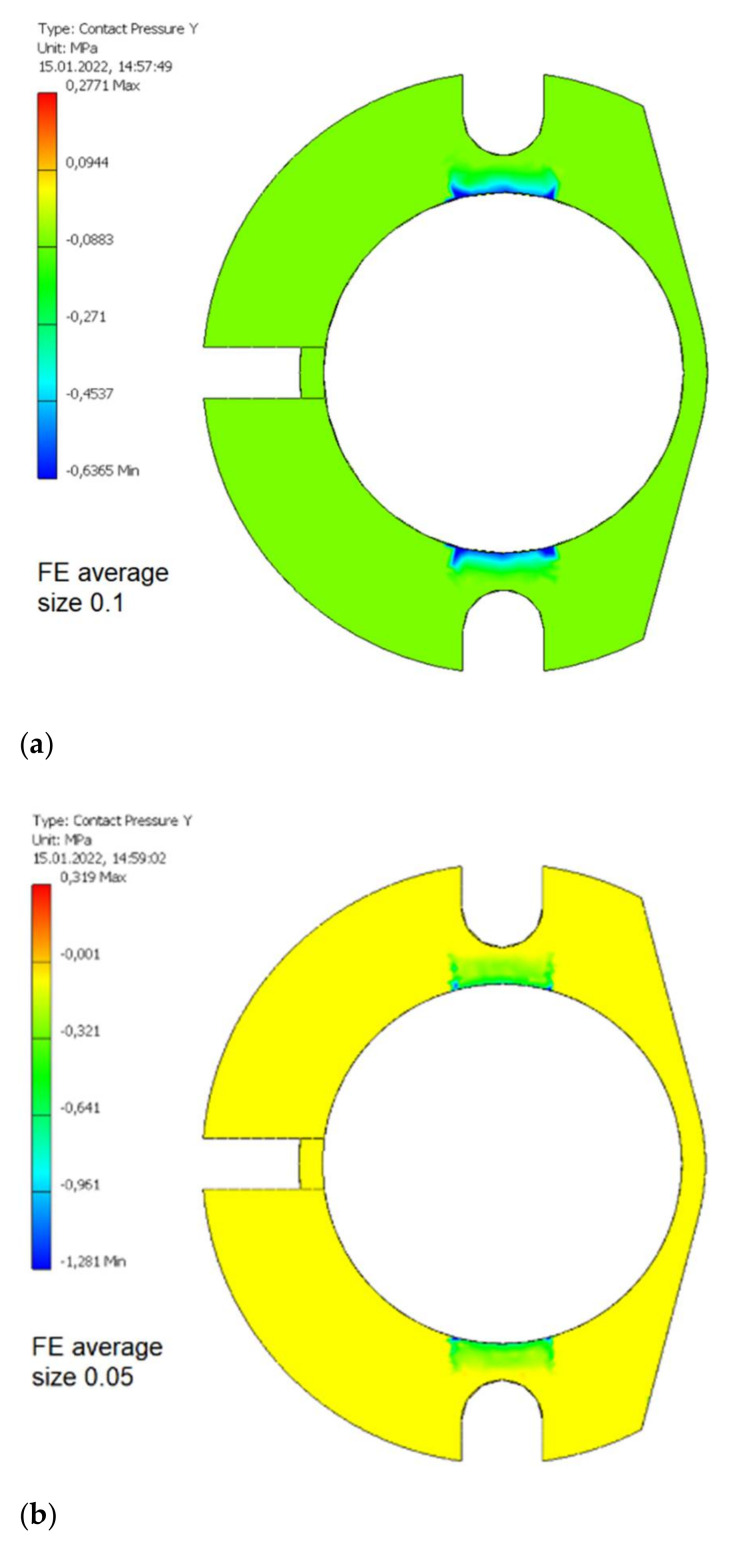

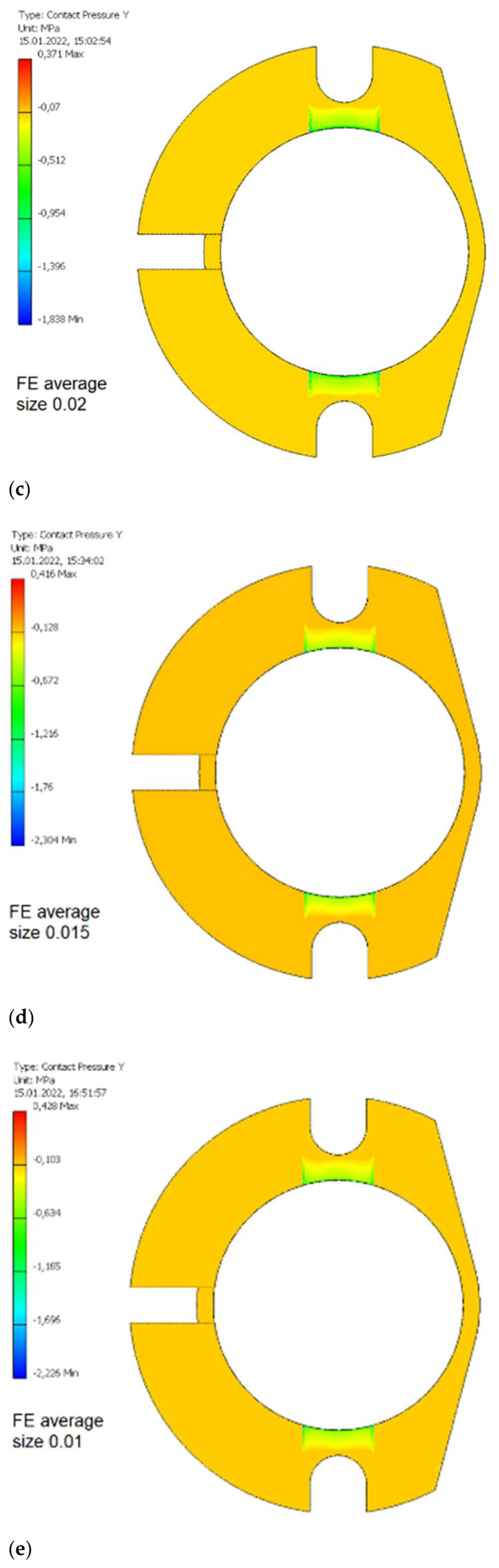
The values of normal contact pressure in the contact zone between the swivel arm and the bobbin case for the various average finite element relative sizes. (**a**) 0.1; (**b**) 0.05; (**c**) 0.02; (**d**) 0.015; (**e**) 0.01.

The obtained values of normal contact pressure in the contact zone between the swivel arm and the bobbin case as a function of average finite element size is presented in Figure 9. The maximum values of such a contact pressure for various average finite element sizes are presented in Table 9. It was assumed that average finite element size equal to 0.015 allow sufficiently accurate values of such a contact pressure to be obtained without unnecessarily increasing computing power and duration.

**Figure 10:**
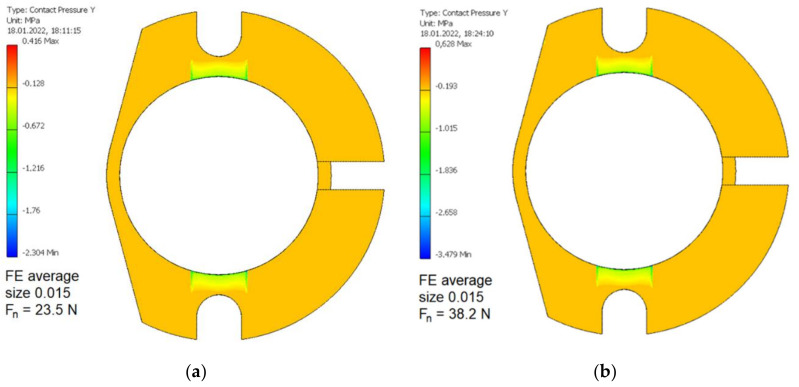
The values of normal contact pressure in two contact zones between the swivel arm and the bobbin case for the average FE relative size equal to 0.015 and two values of the normal force $F_{n}$. (**a**) 25.3 N relating to milling with the course cutter ø4; (**b**) 38.2 N relating to milling with the finishing cutter ø5.

The resulting values of normal contact pressure in two contact zones between the swivel arm and the bobbin case for the average FE relative size equal to 0.015 and two values of the $F_{n}$ are presented in Figure 10. An increase of the normal force $F_{n}$ by 39% resulted in an increase of contact pressure in the mentioned contact zones by 52%.

**Figure 11:**
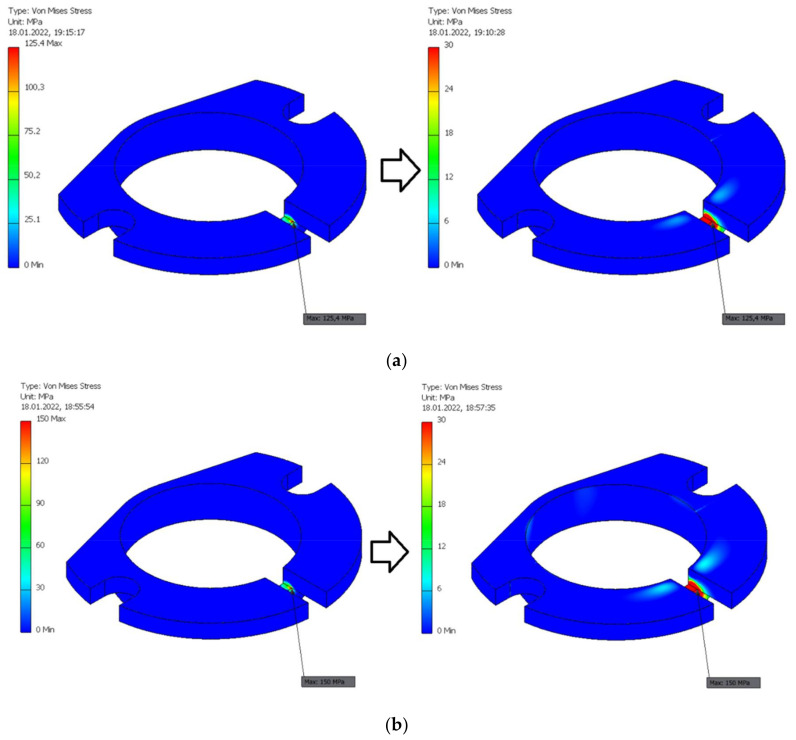
The values of von Mises stresses in the bobbin case milled using various cutters and relative to them, two values of the normal force $F_{n}$. (**a**) 25.3 N relating to milling with the course cutter ø4; (**b**) 38.2 N relating to milling with the finishing cutter ø5.

The resulting values of von Mises stresses in the bobbin case loading by cutting torque for two cases of cutters and relative to them, two values of the normal force $F_{n}$ used are shown in Figure 11. To allow better orientation in the von Mises stress distribution, such a distribution with more limited scale was introduced on the right of each Figure 11 component. In the case of end milling, the maximum value of von Mises stress in the bobbin case material was higher by 20% compared to the case of the coarse milling.

### 5.2. Results of Loading of the Swivel Arm

**Figure 12:**
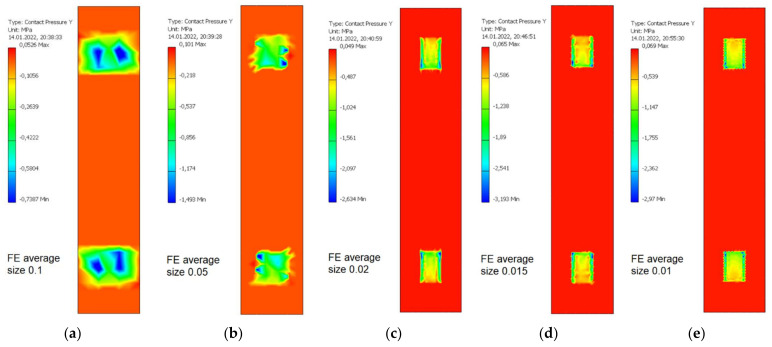
The values of normal contact pressure in the contact zone between eccentric and the segment of swivel arm for the various average finite element relative sizes. (**a**) 0.1; (**b**) 0.05; (**c**) 0.02; (**d**) 0.015; (**e**) 0.01.

The resulting values of normal contact pressure in the contact zone between eccentric and the segment of swivel arm as a function of average finite element size are presented in Figure 12. The maximum values of such a contact pressure for various average finite element sizes are presented in Table 10. It was assumed that average finite element size equal to 0.02 allow sufficiently accurate values of such a contact pressure to be obtained without unnecessarily increasing computing power and duration.

**Figure 13:**
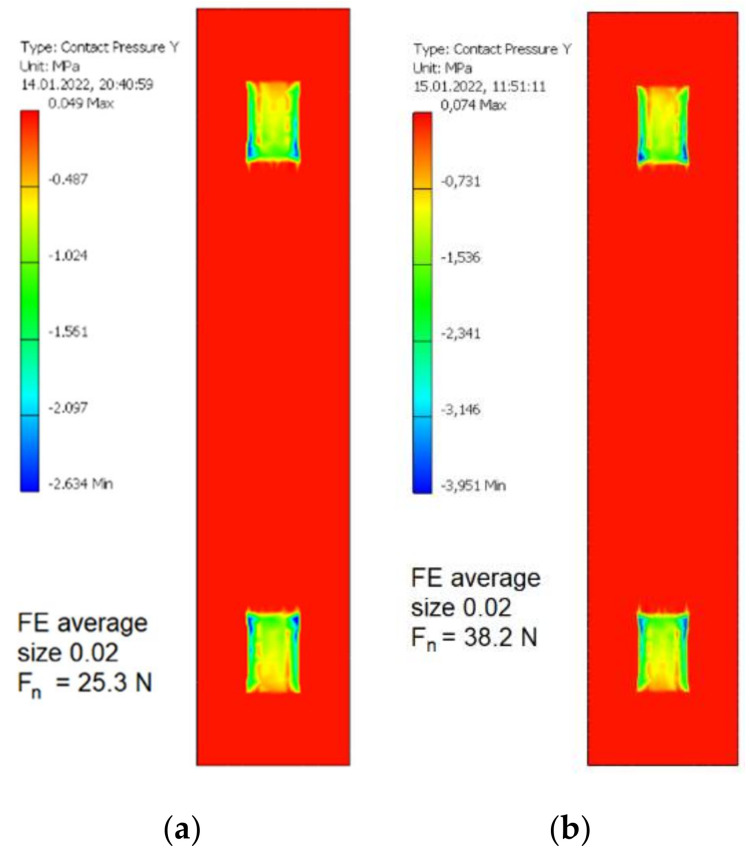
The values of normal contact pressure in two contact zones between eccentric and the segment of swivel arm for the average FE relative size equal to 0.02 and two values of the normal force $F_{n}$. (**a**) 25.3 N relating to milling with the course cutter ø4; (**b**) 38.2 N relating to milling with the finishing cutter ø5.

The resulting values of normal contact pressure in two contact zones between eccentric and the segment of swivel arm for the average FE relative size equal to 0.02 and two values of the $F_{n}$ are presented in Figure 13. An increase of the normal force $F_{n}$ by 39% resulted in an increase of contact pressure in the mentioned contact zones by 50%.

The corresponding values of the total indentation depth δ and distance D are shown in Table 11. It was recommended that for the initial state of the milling chuck, the distance D should be equal at most to 44.9993 mm. Of course, the real values of the deviations of the shapes and positions of the chuck elements are much greater than the calculated values of the total indentation depth δ. This means that the forces generated during the eccentric cam pressing to the swivel arm surface can be much greater than necessary, but at the same time the mating surfaces may wear much faster.

### 5.3. Results of Wear of Elements of the Model of the Eccentric Cam Mating with the Swivel Arm

**Figure 14:**
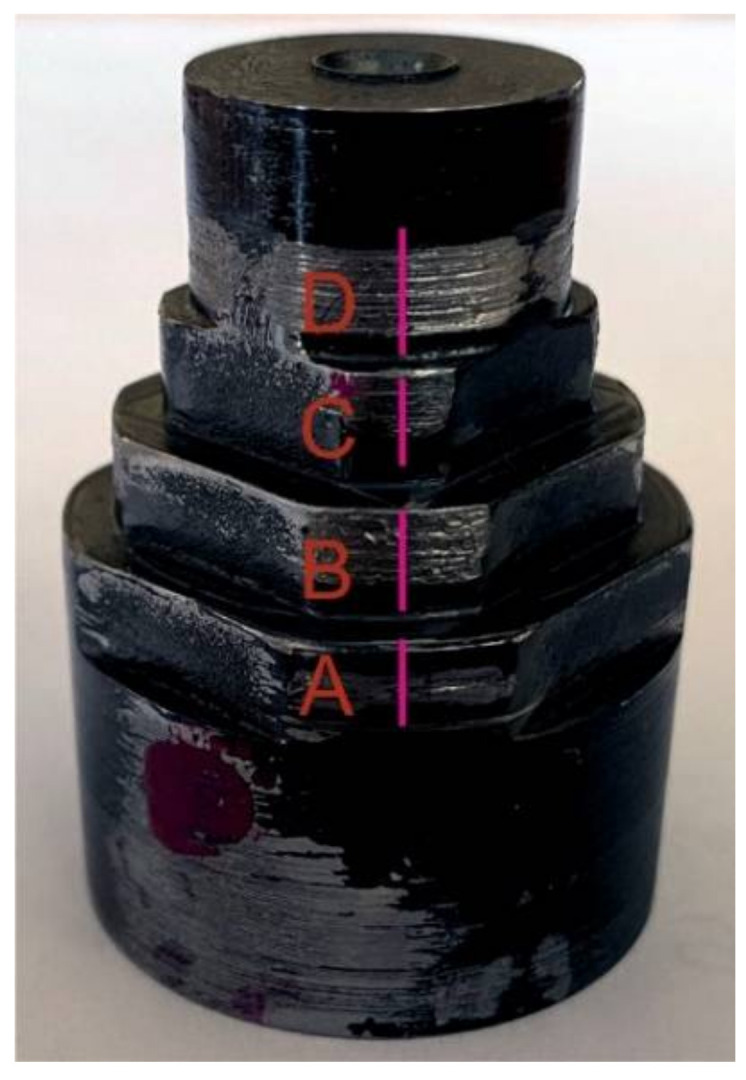
The sample worn initially DLC covered surfaces of steel shaft shoulders after the mating with the front planes of steel plunger fins. A, B, C, D—the worn cylindrical surfaces of the consecutive shaft shoulders.

The obtained views of the sample worn initially DLC covered surfaces of steel shaft shoulders after the mating with the front planes of steel plunger fins are shown in Figure 14. 

**Figure 15:**
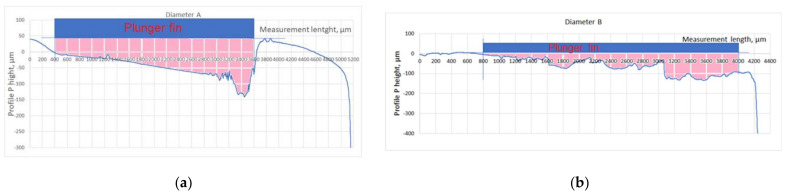

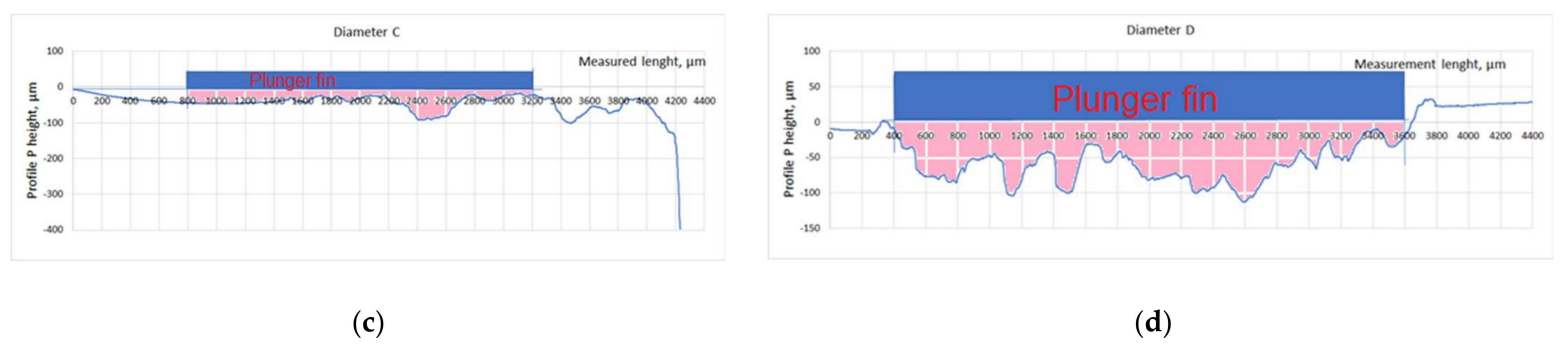
Sample profile of the worn initially DLC covered surfaces of steel shaft shoulders after the mating with the front planes of steel plunger fins. (**a**) Shaft shoulder surface A; (**b**) Shaft shoulder surface B; (**c**) Shaft shoulder surface C; (**d**) Shaft shoulder surface D. Blue rectangle—a schematic view of the fragment of the plunger fin positioned in the operational position during mating with the shaft shoulder cylindrical surface, pink area—the corresponding cross-section area of removed material of the shaft shoulder.

With the step decreasing radius of shaft shoulders, the mean contact pressure values in the relating contact zones between a shaft shoulder and the mating lunger fin enhanced in a step manner. It was due to the same force generated by the spring and loading such contact zones. That was accompanied by the step decrease of slide velocity, as the regime of the angle rotating speed of the shaft was the same. Additionally, the abrasive wear distance decreased in a step manner, respectively. The level of the abrasive removal of the fragments of the DLC protective coatings deposited on the front plane of the steel piston fin and the surface of the steel shaft shoulder was more clearly visible for the higher values of the mean contact pressure in relating contact zones. For the shaft shoulders with two consecutively lowest diameters, the whole DLC protective coatings were practically removed. Interestingly, the accompanying decrease in sliding velocity seemed to less influence the intensity of abrasive removal of the mentioned DLC protective coatings than the increase in contact pressures. Along the axis of the shaft, the profiles of the worn cylindrical surfaces were measured for consecutive shaft shoulders A, B, C, and D, respectively. The obtained courses of such a profile are shown in Figure 15. In each Figure, the blue rectangle represents the fragment of the plunger fin with the front surface perpendicular to the Figure, positioned in the operational position during mating with corresponding shaft shoulder cylindrical surface. Radially it is positioned in the place tangent to the nearest maximal height of the *P* profile registered. The red area represents the corresponding cross-section of a removed material.

The average volumetric wear values for the shaft shoulders are presented in Table 12.

The masses of initial and worn plungers, their mass wear, and volumetric wear are shown in Table 13.

From the obtained values of volumetric wear of shaft shoulders, their linear wear intensity average values were estimated and are presented in Table 14. These values were twice higher than those reported for the dry steel-steel contact obtained on the pin-on-disc tribotester [95] for the 1.7 lower values of contact pressure and the 3-fold higher sliding speed.

**Figure 16:**
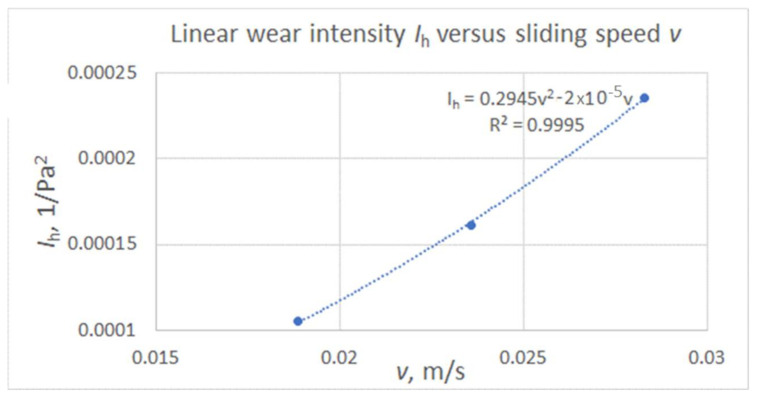
The linear wear intensity average values as a function of sliding speed for shaft shoulders with four cuts.

Such values were expressed as a function of sliding speed for shaft shoulders with four cuts and are presented in Figure 16.

From the obtained values of volumetric wear of plunger fins, the linear wear intensity average values were estimated and are presented in Table 15. They were almost one order higher than those for the corresponding shaft shoulders.

**Figure 17:**
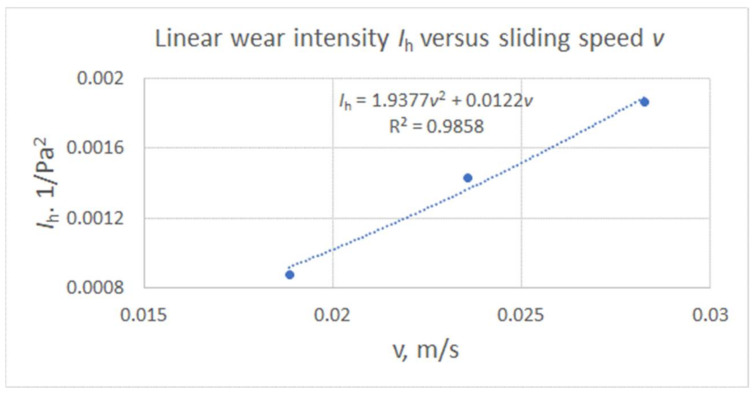
The linear wear intensity average values as a function of sliding speed for plunger fins.

The corresponding values of wear of plunger fin expressed as a function of sliding speed for plunger fins are shown in Figure 17.

It is interesting that the average linear wear intensity of shaft shoulder approximated by the function of sliding speed differed from the average linear wear intensity of the shaft shoulder determined based on measured values of wear from registered *P* profiles only by about 3% for the case of the shaft shoulder with two cuts. Simultaneously, the average linear wear intensity of the plunger fin material approximated by the function of sliding speed differed from the average linear wear intensity of the plunger fin material determined based on measured values of mass wear only by about 6% for the case of the plunger fin mating with the shaft shoulder with two cuts. The loading force, number of cycles, and sliding speed for the case of this shaft shoulder significantly differed from those for the shaft shoulder with four cuts made. Therefore, to some extent, such observation confirms the correctness of the obtained mathematical model for the linear wear intensity characterizing the abrasive wear process of the rotating shaft initially covered with a DLC layer mating with the plunger fin front plane also initially covered with a DLC layer. Such an abrasive wear process proceeded under the conditions of additional impact wear resulting from small dynamic deflections of the tensioned spring affecting the load of the plunger fin front plane.

The values of the wear coefficient $k_{A}$ [mm^2^ N^−1^] described by Ejtehadi et al. [93], as cited in [94], and of the the wear coefficient $k_{A}$ proposed by Watrin et al. [75] for shaft shoulders and corresponding plunger fins are presented in Table 16. The values of the wear coefficient $k_{A}$ [mm2 N−1] described by Ejtehadi et al. [93], as cited in [94], were equal to 0.35–1.18 times of those obtained for the dry steel-steel contact on the pin-on-disc tribotester [95] for the 1.7 lower values of contact pressure and the 3-fold higher sliding speed. For the plunger fin, such values were by about one order higher than those for the corresponding shaft shoulders.

### 5.4. The Calculated Number of Cycles of Opening and Closing of the Milling Chunk until Reaching the Limit Value of the Force Pressing the Bobbin Case during Its Milling

**Figure 18:**
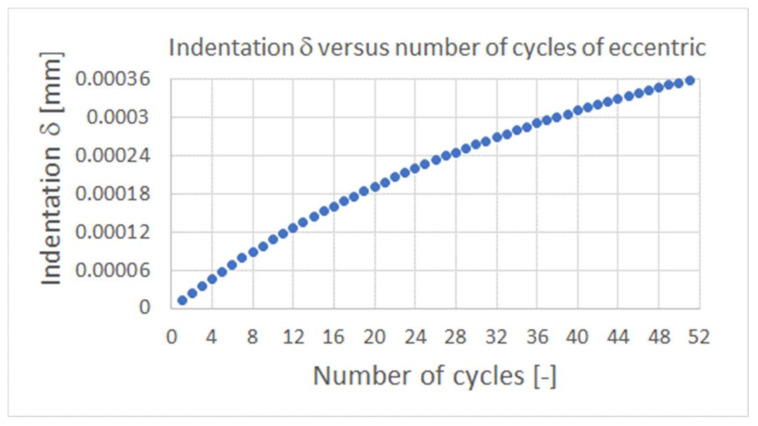
The indentation δ average values as a function of number of cycles of eccentric cam.

The indentation δ versus number of cycles of eccentric is presented in Figure 18. It is visible that after 52 cycles, the indentation δ reached the half initial value of $\delta_{total}$ and the value of force F acting on cylindrical cams of the eccentric will be too small to prevent the micro-displacements of bobbin case during its milling process. The mentioned decrease of indentation δ corresponds to increasing of the distance D above 44.9996 mm due abrasive wear process of mating surfaces. As explained earlier, due to the much greater values of the deviations of the shapes and positions of the chuck elements than the calculated values of the total indentation depth δ, the DLC coated mating surface of the eccentric cams and the swivel arm surface may wear much faster. Therefore, it is necessary to apply correction plates to the upper surface of the swivel arm, cover the cams with a hard repair layer, or replace the entire eccentric.

## 6. Conclusions

From the obtained results some conclusions can be made:

1. The intensity of the abrasive removal of the fragments of the DLC protective coatings deposited on the front plane of the steel plunger fin and the surface of the steel shaft shoulder of cooperating friction with each other under the conditions of non-lubricated contact was higher in the case of higher mean contact pressures. The accompanying reduction in sliding velocity had a lesser effect on the intensity of abrasive wear than the increase in contact pressures.
2. The wear intensities for the plunger fins analyzed were by an order higher than those of corresponding shaft shoulders being below 0.00025 1/Pa^2^ for the sliding speed in contact zone below 0.03 m/s.
3. The linear wear intensities for the plunger fins and the corresponding shaft shoulder may be expressed in terms of a quadratic function of average contact pressure and the polynomial sliding speed in a corresponding contact zone. The forms of the functions of sliding speed were presented in Figure 16 and Figure 17 for shaft shoulders and plunger fin, respectively.
4. The indentation of eccentric cam into mating surface of the swivel arm increased nonlinearly with the increase of number of cycles of the eccentric cam.
5. The use of DLC layer on both the cylindrical cams of the eccentric and the mating surface of swivel arm of the milling chunk provide the needed value of force pressing the milling bobbin case to the milling chunk support only for fifty numbers of opening and closing of the milling chunk.
6. It is necessary to apply the harder protective layer on the surface of the milling chunk, which is more resistive to abrasive wear than the DLC one. The harder protective layer can be obtained using, for example, plasma nitriding process.
7. The conducted tests were under dry conditions and low sliding speed values below 0.03 m/s and contact pressure. Therefore, further investigations can be carried out for lubrication with various lubricants and for higher values of sliding speed and contact pressure in contact zone between mating surfaces of elements made of steel.

## Figures and Tables

**Table 1:** Parameters of the end milling process for the two cutters used.

Parameter	Course Cutter ø4	Finishing Cutter ø5
Rotational speed n [rpm]	4200	4000
Cutting speed $V_{f}$ [mm/min]	500	600
Cutting depth b [mm]	0.5	0.5
Feed per revolution $f_{n}$ [mm/rev]	0.019	0.15

**Table 2:** Values of feed per tooth $f_{z}$ for the course and finishing milling cutter.

Parameter	Coarse Cutter ø4	Finishing Cutter ø5
$f_{z}$ [mm/t]	0.039	0.05

**Table 3:** Values of cutting are $A_{D}$ for the course and finishing milling cutter.

Parameter	Coarse Cutter ø4	Finishing Cutter ø5
$A_{D}$ [mm^2^]	0.0195	0.025

**Table 4:** The determined values of $k_{c}$ and $F_{c}$ for the two cutters used.

Parameter	Course Cutter ø4	Finishing Cutter ø5
Specific cutting resistance $k_{c}$ [N/mm^2^]	3720	3470
Circumferential force component $F_{c}$ [N]	72.54	86.75

**Table 5:** The determined values of the tangential pressure $p_{t}$ for the two cutters used.

Parameter	Course Cutter ø4	Finishing Cutter ø5
Tangential pressure $p_{t}$ [MPa]	0.56	0.85

**Table 6:** The determined values of the normal force $F_{n}$ for the two cutters used.

Parameter	Course Cutter ø4	Finishing Cutter ø5
Normal force $F_{n}$ [N]	25.3	38.2

**Table 7:** The parameters of the compression spring.

Parameter	Unit	Value
Total number of coils	$n_{t}$ [-]	28
Number of active coils	n [-]	26
Free spring length	$L_{0}$ [mm]	90
External diameter	$D_{e}$ [mm]	8
Internal diameter	$D_{i}$ [mm]	6
Average diameter	D [mm]	7
Wire diameter	d [mm]	1
Material modulus of elasticity for steel	G [MPa]	80,000
Spring rate	R [N/mm]	1.12

**Table 8:** The spring deflections and the respective forces loading the contact zone between the movable plunger 2 and the shaft shoulder 1 (Figure 7).

Parameter	Unit	Value
Minimal initial operational spring deflection	$f_{min}^{init}$ [mm]	42
Minimal end operational spring deflection	$f_{min}^{end}$ [mm]	42.3
Maximal initial operational spring deflection	$f_{max}^{init}$ [mm]	50
Maximal end operational spring deflection	$f_{max}^{end}$ [mm]	50.5
Force related to the minimal initial operational spring deflection	$F_{min}^{init}$ [N]	47
Force related to the minimal end operational spring deflection	$F_{min}^{end}$ [N]	47.3
Force related to the maximal initial operational spring deflection	$F_{max}^{init}$ [N]	56
Force related to the maximal end operational spring deflection	$F_{max}^{end}$ [N]	56.6

**Table 9:** The maximum values of normal contact pressure in the contact zone between the swivel arm and the bobbin case for various average finite element sizes.

Average Relative Size of Finite Element [-]	Maximum Contact Pressure in the Contact Zone between the Bobbin Case and the Swivel Arm Loaded by the Normal Force $F_{n}$ = 25.3 N for Milling with the Course Cutter ø4 [MPa]
0.1	0.64
0.05	1.28
0.02	1.84
0.015	2.30
0.01	2.23

**Table 10:** The maximum values of normal contact pressure in the contact zones between eccentric and the segment of swivel arm for various average finite element sizes.

Average Relative Size of Finite Element [-]	Maximum Contact Pressure in The Contact Zones between Eccentric and the Segment of Swivel Arm Loaded by the Normal Force $F_{n}$ = 25.3 N for Milling with the Course Cutter ø4 [MPa]
0.1	0.74
0.05	1.49
0.02	2.63
0.015	3.19
0.01	2.97

**Table 11:** The values of the total indentation depth δ and distance D for the two cutters used.

Parameter	Course Cutter ø4	Finishing Cutter ø5
Total indentation depth δ [µm]	0.5	0.5
Distance D [mm]	44.9995	44.9993

**Table 12:** The average volumetric wear values for the shaft shoulders.

Shaft Shoulder Symbol	Average Volumetric Wear [mm^3^]	Average Contact Pressure [MPa]	Sliding Speed [m/s]
A	1.11 ± 0.65	3.224	0.028
B	5.49 ± 0.42	3.201	0.024
C	2.31 ± 0.27	3.157	0.019
D	7.03 ± 0.58	3.722	0.016

**Table 13:** The masses of initial and worn plungers, mass wear, and volumetric wear.

Plunger Symbol	Mass of a New Plunger [g]	Mass of A Worn Plunger [g]	Mass Wear [g]	Volumetric Wear [mm^3^]
A	5.47 ± 0.05	4.79 ± 0.05	0.68 ± 0.1	87.18
B	5.48 ± 0.05	5.10 ± 0.05	0.38 ± 0.1	48.72
C	5.48 ± 0.05	5.33 ± 0.05	0.15 ± 0.1	19.23
D	5.51 ± 0.05	5.52 ± 0.05	0.46 ± 0.1	58.97

**Table 14:** The linear wear intensity values for the shaft shoulders.

Shaft Shoulder Symbol	Average Linear Wear Intensity [1/Pa^2^]	Average Linear Wear Intensity Approximated by the Function of Sliding Speed [1/Pa^2^]	Differences [%]
A	0.000235553	0.000234869	0.3
B	0.000161278	0.000163025	1.1
C	0.000105323	0.000104261	1.0
D	8.21676 × 10^−5^	7.97833 × 10^−5^	2.9

**Table 15:** The linear wear intensity values for the plunger fins.

Plunger Symbol.	Average Linear Wear Intensity [1/Pa^2^]	Average Linear Wear Intensity Approximated by the Function of Sliding Speed [1/Pa^2^]	Differences [%]
A	0.001866855	0.001894018	1.5
B	0.001430812	0.001363199	4.7
C	0.000877067	0.000918441	4.7
D	0.000688962	0.000728333	5.7

**Table 16:** The values of wear coefficient $k_{A}$ [mm^2^ N^−1^] described by Ejtehadi et al. [93], as cited in [94], and of the wear coefficient $k_{A}$ proposed by Watrin et al. [94] for the shaft shoulders and corresponding plunger fins.

Shaft Shoulder/Plunger Fin Symbol	Wear Coefficient $k_{A}$ [mm^2^ N^−1^] Described by Ejtehadi et al.		Wear Coefficient $k_{A}$ Proposed by Watrin et al.
	Shaft shoulder	Plunger Fin	
A	7.30652 × 10^−5^	0.000579071	3.05995 × 10^−7^
B	5.03867 × 10^−5^	0.000447018	3.69062 × 10^−7^
C	3.33648 × 10^−5^	0.000277843	4.80238 × 10^−7^
D	2.20742 × 10^−5^	0.000185088	2.19554 × 10^−7^

## Data Availability

Not applicable.
